# The Relationship Between Coronary Dominance and Positive Results in Myocardial Perfusion Imaging

**DOI:** 10.7759/cureus.27343

**Published:** 2022-07-27

**Authors:** Mathhar Aldaoud, Hardik Patel, Jacob Alex, Yousif bader, Marcel Zughaib

**Affiliations:** 1 Interventional Cardiology, Ascension Providence Hospital, Michigan, USA; 2 Cardiology, Ascension Providence Hospital, Southfield, USA; 3 Internal Medicine, Ascension Providence Hospital, Southfield, USA; 4 Cardiovascular Medicine, Ascension Providence Hospital, Southfield, USA

**Keywords:** stable ischemic heart disease, coronary dominance, cad: coronary artery disease, coronary artery angiogram, nuclear stress test

## Abstract

Objective

Our study aims to evaluate the possible relationship between coronary artery dominance and its effect on accurately identifying reversible ischemia of inferior/inferior-lateral wall on cardiac perfusion imaging.

Background

Coronary artery dominance is conventionally defined by the vessel which gives the rise to the AV nodal artery/posterior descending artery (PDA). Previous studies have explored the potential effect of coronary dominance on the accuracy of single photon emission computed tomography (SPECT) myocardial perfusion imaging (MPI) tests in detecting inferior/inferior-lateral wall ischemia; further evidence is necessary to study that potential effect.

Methods

We conducted a single-center retrospective analysis to explore the potential relationship between coronary artery dominance and inferior/inferior-lateral wall ischemia on SPECT imaging. We identified a cohort of patients with a reversible defect(s) in the inferior and/or inferolateral walls on SPECT MPI who had subsequently undergone invasive coronary angiography. Coronary angiography was used to determine coronary dominance and to confirm the presence/absence of obstructive coronary artery disease in the distribution of the inferior and/or inferolateral wall(s). We correlated the findings on SPECT MPI to coronary angiography to identify true positives and false positive MPIs.

Results

A cohort of 200 patients was identified, patients in the cohort had undergone stress MPI with reversible defects with subsequent invasive coronary angiography. Baseline characteristics including age, BMI and sex were fairly well-balanced between the groups. The mean age was 68 +/- 11 in the right dominant group and 70 +/- 9 in the non right dominant group. One hundred and sixty-one patients (81%) were found to have right dominant circulation and 39 patients (19%) were found to have left or codominant circulation. Of the 161 patients in the right dominant group, 58 patients (36%) were found to have false positive stress MPI. Of the 39 patients in the left or codominant group, 23 patients (59%) were found to have false positive stress MPI. The incidence of false positive stress MPI in the inferior and inferolateral distribution is significantly higher in patients with non-right dominant coronary anatomy (p-value: 0.01).

Conclusion

Non-right coronary dominant anatomy could have high false positive MPI results in the inferior and inferolateral distribution. Therefore, the interpreting clinicians should exercise caution during the clinical evaluation of these patients.

## Introduction

Coronary artery dominance is conventionally defined by the vessel which gives rise to the AV nodal artery/posterior descending artery (PDA). The PDA runs along the posterior interventricular groove towards the apex of the heart, supplying the inferior wall of the heart and the AV node [1]. Approximately 70%-80% of the general population are right dominant, 5%-12% are left dominant and the remainder is codominant [2]. Previous studies have explored the potential effect of coronary dominance on the accuracy of single photon emission computed tomography (SPECT) myocardial perfusion imaging (MPI) tests in detecting inferior/inferior-lateral wall ischemia, Ali et al. showed more false positive MPI results were noted in non-right dominant circulation in detecting inferior/inferior-lateral wall ischemia [3]. However, more studies are needed to confirm those findings. Our study aims to evaluate the possible relationship between coronary artery dominance and its effect on accurately identifying reversible ischemia of inferior/inferior-lateral wall on perfusion imaging.

## Materials and methods

Methods

We conducted a single-center retrospective analysis to explore the potential relationship between coronary artery dominance and inferior/inferior-lateral wall ischemia on SPECT imaging. The study protocol was approved by the local Institutional Review Board (IRB). We reviewed SPECT MPI stress tests performed over one year period from January 2018 to December 2019. Patients with prior coronary artery bypass grafting were excluded. We identified a cohort of patients with a reversible defect(s) in the inferior and/or inferolateral walls on SPECT MPI who had subsequently undergone invasive coronary angiography. 

All patients underwent one day of 99mTechnitium-sestamibi rest/stress SPECT MPI study, electrocardiography-gated MPI protocol. Exercise and pharmacological MPIs were performed on a SPECT scanner (Siemens SPECT dual head camera). Exercise and pharmacologic testing were physician-supervised and performed within the guidelines of the American College of Cardiology/American Heart Association. Imaging was carried out within the guidelines of the American Society of Nuclear Cardiology. The method of stress was an exercise in 22% of patients, and a vasodilator (Regadenoson) in 78%, p-value of 0.72 between the two groups.

Coronary angiography was used to determine coronary dominance and to confirm the presence/absence of obstructive coronary artery disease in the distribution of the inferior and/or inferolateral wall(s). Obstructive CAD angiographically was defined as a greater than or equal to 75% luminal stenosis requiring revascularization or 50%-75% stenosis with abnormal hemodynamic assessment (FFR/iFR) [4]. The need for hemodynamic confirmation in the catheterization laboratory was based on the judgment of the operator at the time of angiography. We correlated the findings on SPECT MPI to coronary angiography to identify true positives and false positive MPIs. True positives were defined as patients with inferior/inferior-lateral wall ischemia on MPI and significant obstructive stenosis of the epicardial vessel supplying that territory on coronary angiography or via hemodynamic assessment. False positives were defined as inferior/inferior-lateral wall ischemia on MPI with non-obstructive/no CAD on invasive angiography or via hemodynamic assessment. We performed statistical analysis to evaluate the possible effect of coronary artery dominance on the detection of inferior/inferior-lateral wall ischemia on perfusion imaging. The cut-off p-value for statistical significance was less than 0.05. Fisher's test and t-test were used to compare the two groups. Baseline characteristics were collected including gender, age, body mass index, history of CAD, diabetes mellitus, hypertension and dyslipidemia.

## Results

A cohort of 200 patients was identified who had undergone stress MPI with reversible defects with subsequent invasive coronary angiography. Baseline characteristics including age, BMI and sex were fairly well-balanced between the groups (Table 1). The mean age was 68 +/- 11 in the right dominant group and 70 +/- 9 in the non-right dominant group. One hundred sixty-one patients (81%) were found to have right dominant circulation and 39 patients (19%) were found to have left or codominant circulation. Of the 161 patients in the right dominant group, 58 patients (36%) were found to have false positive stress MPI. Of the 39 patients in the left or codominant group, 23 patients (59%) were found to have false positive stress MPI (Table 2). The incidence of false positive stress MPI was statistically significant between the right dominant and non-right dominant groups (p-value: 0.01).

**Table 1:** Baseline characteristics

Baseline characteristics	Right dominant	Left Dominant or Codominant	P-value
Male gender	102/161 (63%)	23/39 (58%)	0.7127
Age (years)	68 +- 11	70.6 +- 9	0.9098
History of Hypertension (mm Hg)	128/161 (80%)	33/39 (86%)	0.6525
History of coronary artery disease	48/161 (29%)	6/39 (15%)	0.0737
History of diabetes mellitus	32/161 (20%)	11/39(28%)	0.279
History of dyslipidemia	61/161 (38%)	13/39 (33%)	0.71
Body mass index	29 +- 3.3	30.4 +- 5.5	0.846

**Table 2:** Differences in false positive rates in the right dominant group vs. the left or codominant group (36% vs 59%, respectively) p-value of 0.01.

Coronary dominance	False positive	True positives	Total
Right Dominant (patients)	58 (36%)	103 (64%)	161
Left Dominant or Codominant (patients)	23 (59%)	16 (41%)	39
Total	81 (41%)	119 (59%)	200

## Discussion

SPECT MPI remains a widely used modality to analyze myocardial perfusion with a sensitivity of 0.83 and a specificity of 0.77 according to a meta-analysis conducted by Marcus et al. [5]. MPI plays an important role in clinical decision-making as a gatekeeper for further intervention and has been proven to be a cost-effective non-invasive imaging modality to evaluate for the presence of obstructive coronary artery disease [3]. However, nuclear perfusion imaging is subject to a variety of artifacts and pitfalls, which may affect its clinical utility. Coronary dominance was previously suggested as a factor that affects the accuracy of the SPECT MPI imaging. Our study demonstrated additional evidence that SPECT MPI in detecting inferior/inferior-lateral wall ischemia has a statistically significant higher incidence of false positive studies with left or codominant coronary circulation compared to its right dominant counterpart. This finding was previously described in an observational study in which the authors hypothesized that the flow tracer in a left dominant or codominant circulation may show relatively decreased uptake in the inferior wall that might not be indicative of flow-limiting stenosis [3]. In addition, anatomical variation in vessel size might also be contributing to this observed phenomenon [3].

In our study, 81% of patients were right dominant vs 19% left or codominant. This is consistent with and representative of the coronary artery dominance distribution found in the general population -- and supports the strength of our study population in representing the general population.

The incidence of false positive studies in the group with right coronary artery dominance was relatively high (39%) in comparison to the reported false positive rate in other studies [5]. This can be explained by a larger male population in the study cohort. It is known that inferior wall artifacts are more common in males due to diaphragmatic attenuation [5]. Prone imaging has been shown to improve inferior wall attenuation artifacts [6,7]; however, the supine-prone acquisition protocol is underutilized at our institution. Despite the high baseline incidence of false positives in the group with right coronary artery dominance, there was a statistically significant difference in the incidence of false positive studies in the left or codominant circulation (59%) -- an absolute difference of 20% with a p-value of 0.01. The marked difference in the false positive rates illustrates the possible relationship between coronary artery dominance and inferior wall attenuation artifacts. Understanding and incorporating this relationship into daily practice in patients with inferior wall attenuation artifacts, when coronary dominance was previously established with imaging, may improve the reader's acumen and potentially reduce the need for downstream testing.

Limitation

The study population included only 200 patients. However, for the purpose of our study, the cohort was a fair representation of the general population as pertaining to the distribution of coronary artery dominance. The study did not include patients with negative test results; therefore, sensitivity and specificity could not be ascertained.

## Conclusions

The accuracy of SPECT MPI to detect inferior/inferior-lateral wall ischemia is affected by coronary artery dominance. In our study, the non-right dominant patients were noted to have higher false positive stress MPI results in detecting inferior/inferior-lateral wall ischemia. More studies are needed to explain this phenomenon.
